# Altered periaqueductal gray resting state functional connectivity in migraine and the modulation effect of treatment

**DOI:** 10.1038/srep20298

**Published:** 2016-02-03

**Authors:** Zhengjie Li, Mailan Liu, Lei Lan, Fang Zeng, Nikos Makris, Yilin Liang, Taipin Guo, Feng Wu, Yujie Gao, Mingkai Dong, Jie Yang, Ying Li, Qiyong Gong, Fanrong Liang, Jian Kong

**Affiliations:** 1 The 3rd Teaching Hospital, Chengdu University of Traditional Chinese Medicine, Chengdu, Sichuan, China; 2 Department of Traditional Chinese Medicine, Medical College of Xiamen University, Xiamen, Fujian, China; 3 Acupuncture & Tuina School, Hunan University of Chinese Medicine, Changsha, Hunan, China; 4 Department of Psychiatry, Massachusetts General Hospital and Harvard Medical School, Charlestown, MA, USA; 5 Martinos Center for Biomedical Imaging, Massachusetts General Hospital, Charlestown, MA, USA; 6 Neuroscience Program, Wellesley College, Wellesley, MA, USA; 7 Huaxi MR Research Center, West China Hospital of Sichuan University, Chengdu, Sichuan, China

## Abstract

The aims of this study were to 1) compare resting state functional connectivity (rs-fc) of the periaqueductal gray (PAG), a key region in the descending pain modulatory system (DPMS) between migraine without aura (MwoA) patients and healthy controls (HC), and 2) investigate how an effective treatment can influence the PAG rs-fc in MwoA patients. One hundred MwoA patients and forty-six matched HC were recruited. Patients were randomized to verum acupuncture, sham acupuncture, and waiting list groups. Resting state fMRI data were collected and seed based functional connectivity analysis was applied. Compared with HC, MwoA patients showed reduced rs-fc between the PAG and rostral anterior cingulate cortex/medial prefrontal cortex (rACC/mPFC), key regions in the DPMS and other pain related brain regions. The reduced rs-fc between the PAG and rACC/mPFC was associated with increased migraine headache intensity at the baseline. After treatments, rs-fc between the PAG and the rACC in MwoA patients significantly increased. The changes of rs-fc among the PAG, rACC and ventral striatum were significantly associated with headache intensity improvement. Impairment of the DPMS is involved in the neural pathophysiology of migraines. Impaired DPMS in migraine patients can be normalized after effective treatment.

Migraine is a common chronic brain disorder with prevalence of 18.2% among females and 6.5% among males in the United States^1^. About 64% of migraine patients are migraine without aura (MwoA) subtype^2^. Migraine has become an important public health and social issue due to its high prevalence, large medical burden^3^, disabling effects^4^ and serious reduction in quality of life^5^. Nevertheless, many questions remain regarding the pathophysiology and factors that influence this complex disorder. An improved understanding of the mechanisms underlying migraine and its modulators/treatment will open new and promising avenues for discovering the causes of migraine and developing new therapeutic targets.

Current concepts of migraine suggest that the disturbed homeostasis of the trigeminovascular nociceptive pathway is a key factor for susceptibility to migraine headache. The pathophysiology of an imbalance in activity between the brainstem nuclei regulating antinociception and vascular control, especially in the ventrolateral periaqueductal gray (vlPAG) has been proposed^6^,^7^,^8^. Animal studies also showed that descending modulation of the trigeminocervical complex (TCC), through the vlPAG and rostral ventromedial medulla (RVM), could cause the activation of ‘on’ cells and the inhibition of ‘off’ cells in the RVM, which seems to be critical for activation of TCC and development of migraine headache^9^,^10^.

Despite the potential importance of the descending pain modulation system (DPMS) in the development of migraine^9^, investigating the functional status of DPMS non-invasively in humans remains a challenge. The resting-state fMRI technique allows for identification of correlations during rest between remote brain regions (resting-state functional connectivity, rs-fc) through their highly correlated low-frequency spontaneous fluctuations non-invasively^11^. Recently, we started to apply resting state functional connectivity (rs-fc) to investigate the DPMS. In an early study, we investigated the rs-fc of PAG, a key region in the DPMS, and found significant rs-fc among the PAG, the central region of the DPMS, the rACC, the rostroventral medulla (RVM), the orbital prefrontal cortex and other brain regions^12^. These findings demonstrate the feasibility of using rs-fc to non-invasively investigate the DPMS in humans. More recently, studies from our group^13^,^14^ and other groups^15^,^16^ further endorsed the value of PAG rs-fc in studying chronic pain disorders such as migraine^15^,^16^ and chronic low back pain.^13^

Thus, in this study, we first compared DPMS rs-fc between migraine patients (during interictal period when the patients are free from headache symptoms) and matched healthy controls. We then explored if an effective non-pharmacological therapeutic modality, longitudinal acupuncture treatments, could modulate the rs-fc of DPMS. We hypothesize that 1) migraine is associated with dysfunction of DPMS as indicated by rs-fc changes between the PAG and key regions of DPMS; 2) an effective treatment can normalize the impaired rs-fc of DPMS.

We choose acupuncture as treatment in this study because previous studies have found that longitudinal acupuncture can significantly reduce the symptoms of migraineurs^17^,^18^. Nevertheless, it is important to note that this study focuses on how the PAG rs-fc can be modified after an effective treatment and its association with clinical outcomes, not on the mechanism of acupuncture treatment, which require significant clinical improvement as compared with sham treatment, and require a much larger sample size.

## Methods

### Participants

The study protocol was approved by the Ethics Committee of the 1st Teaching Hospital of Chengdu University of Traditional Chinese Medicine. The experiment was performed in accordance with approved guidelines. Participants were enrolled from the outpatient department of the 3rd Teaching Hospital and the campus of Chengdu University of Traditional Chinese Medicine. This study was registered on www.clinicaltrial.gov (NCT01152632, June 27, 2010). Informed consent was obtained from all subjects. The recruitment started in June 2011 and ended in November 2013.

Migraine without aura patients were included in this study. Diagnosis of MwoA was based on the International Classification of Headache Disorders, 2nd Edition ICHD-II MwoA criteria^19^. Inclusion criteria required that all patients: (1) were aged between 17–45 years and right-handed, (2) had not taken any prophylactic headache medicine, or acupuncture treatment during the last three months, (3) had at least six months of migraine duration, and (4) had at least one headache attack per month during the last three months. Patients were excluded if they: (1) were alcohol or drug abusers, (2) were pregnant or during lactation, (3) suffered from psychiatric, neurologic, cardiovascular, respiratory or renal illnesses, (4) suffered from any other chronic pain conditions or had a history of head trauma, with loss of consciousness, (5) had MRI contraindications, such as claustrophobia, and (6) had acupuncture contraindications, such as bleeding tendency.

Right-handed healthy controls (HC), aged between 17–45 years, free from any chronic pain conditions were recruited for this study as healthy controls. Each subject underwent a review of medical history evaluation, physical examination, hepatic function, renal function, and routine analysis of blood, urine, and stool to exclude organic disease carriers. Individuals with abnormal test results or a history of head trauma with loss of consciousness, pregnancy or lactation were excluded.

### Study Design

**Figure 1:**
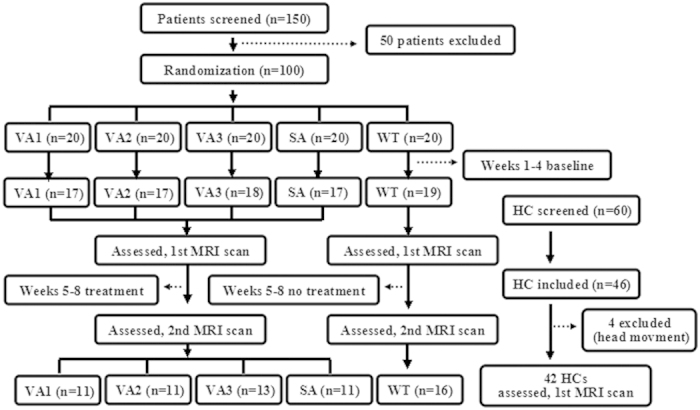
Study flow chat. HC, healthy controls; VA, verum acupuncture; SA, sham acupuncture; WT, waiting-list.

The total observation period for MwoA patients of this study was eight weeks. After screening, all MwoA patients were randomized into five groups, including verum acupuncture groups 1, 2, 3 (VA1, VA2, VA3), sham acupuncture group (SA) and waiting-list (WT) group. Weeks one to four served as baseline phase were used to record baseline headache diaries. Weeks five to eight were served as intervention phase, during which patients in treatment groups received verum or sham acupuncture treatment. All patients continued recording headache diaries during this period. In addition, MRI scans were applied at the end of the forth and eighth weekends for migraine patients. All MwoA patients were migraine-free for at least 72 hours at the time of MRI scan. HC received only the baseline MRI scan (Fig. 1).

### Interventions

**Figure 2:**
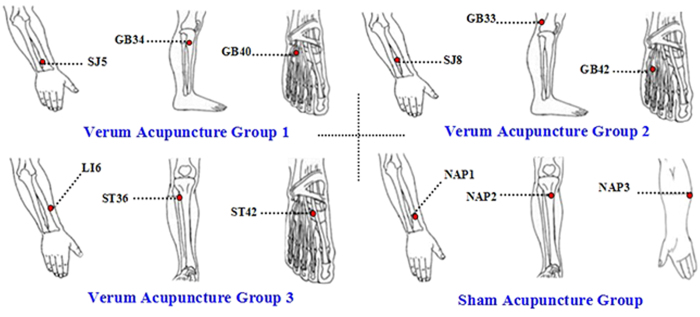
Acupoints location.

Acupoint and non-acupoint selection were similar to those in our previous RCT studies^18^,^20^. Acupoints selected in VA1 were Yanglingquan (GB34), Qiuxu (GB40) and Waiguan (SJ5). VA2 acupoints were Xiyangguan (GB33), Diwuhui (GB42) and Sanyangluo (SJ8). VA3 acupoints were Zusanli (ST36), Chongyang (ST42) and Pianli (L16). SA acupoints included NAP1, NAP2 and NAP3 (Fig. 2).

Two licensed acupuncturists administrated all acupuncture treatment. All acupoints and non-acupoints were punctured bilaterally using disposable stainless steel fine needles. The needles were inserted perpendicularly at a penetration of 5 to 15 mm and were gently twisted, lifted, and thrust at an even amplitude, force, and speed to acquire deqi sensation (deqi sensation is a complex feeling including soreness, numbness, heaviness, distention or dull pain at the site of needle placement) in all treatment groups^21^. The MwoA patients in acupuncture groups received 20 treatments (30 min each) over a four-week period: once per day for five weekdays followed by a two-day break. No acupuncture or sham acupuncture treatments were applied on healthy controls and migraines in waiting-list group.

Patients were instructed and also agreed not to take any regular medications for the treatment of migraines. In cases of severe pain, ibuprofen (300 mg each capsule with sustained release) was allowed as rescue medication.

### Outcome Measures

The primary outcome of this study was the PAG rs-fc change. The clinical outcome were the headache intensity (using a 0–10 visual analogue scale (VAS), 0 indicates no pain, 10 indicates worst pain ever) and frequency of migraine attacks (the number of migraines separated by pain free intervals of at least 48 hours of headache) based on patients’ headache diary according to the guidelines of the IHS for Clinical Trials in Migraine^22^. In addition, self-rating anxiety scale (SAS) and self-rating depression scale (SDS) were applied to assess the MwoA patients’ anxiety and depression status^23^,^24^.

### MRI data acquisition

MRI data was acquired with a 3.0T magnetic resonance scanner (Siemens 3.0T Trio Tim, Munich, Germany) with an 8-channel phase-array head coil at the West China Hospital MRI center. Subjects were asked to stay awake and to keep their heads still during the scan, with their eyes closed and ears plugged. Prior to the functional run, a high-resolution structural image for each subject was acquired using a three-dimensional MRI sequence with a voxel size of 1 mm^3^ employing an axial fast spoiled gradient recalled sequence (TR = 1900 ms; TE = 2.26 ms; data matrix, 256 × 256; field of view, 256 × 256 mm^2^). The BOLD resting-state functional images were obtained with echo-planar imaging (30 contiguous slices with a slice thickness of 5 mm; TR = 2000 ms; TE = 30 ms; flip angle, 90°; field of view, 240 × 240 mm^2^; data matrix, 64 × 64; total volumes, 180).

### Data Analysis

#### Clinical data analysis

The clinical outcomes were analyzed using SPSS16.0 (SPSS Inc, Chicago, IL). Within and between-group comparisons were performed using paired or unpaired *t*-tests or χ^2^, as appropriate. The significant level used for the statistical analysis with 2-tailed testing was 5%. Continuous variables were presented as the mean with 95% confidence intervals (CI). Categorical variables were described as n (percentage).

#### PAG seed based functional connectivity analysis

All pre-processing steps and the rs-fc analysis were performed using DPARSFA^25^ (Data Processing Assistant for Resting State fMRI, Advanced) based on SPM8 (http://www.fil.ion.ucl.ac.uk/spm). The first ten volumes were not analyzed to allow for signal equilibration effects. The fMRI images were slice timing corrected, head motion corrected, coregistered to respective structural images for each subject, segmented, regressed out 6 head motion parameters, white matter signal and cerebrospinal fluid (CSF) signal, normalized by using structural image unified segmentation, and then re-sample to 3-mm cubic voxels. Subjects with head movements exceeding 2 mm on any axis or head rotation greater than 2° were excluded. Similar to our previous study^13^, we removed frames with FD >0.5 mm (‘scrubbing’), one time point before ‘bad’ time points and two time points after ‘bad’ time points were deleted. The data was then detrended, bandpass filtered from 0.01 to 0.08 Hz and smoothed with a 6 mm full-width half-maximum (FWHM) Gaussian kernel.

We chose the right vlPAG (x = 4, y = -26, z = -14, with 3 mm radius) as the seed. The rationales for choosing this seed are: 1) we found that increased levels of heat pain can evoke a significant fMRI signal increase in this region^26^, 2) it is located within the vlPAG, which is believed to be important for opioid antinociception^27^, and 3) in previous rs-fc studies, we found the seed is functionally connected to the key regions of DPMS during resting in healthy subjects^12^, and showed significant difference between healthy subjects and chronic low back pain patients^13^.

Given that the PAG seed region sits adjacent to a ventricle with significant pulsatile effect, we also chose a seed from ventricular aqueduct nearby the PAG seed (x = 0, y = −34, z = −12, with 3 mm radius) and seeds from the forth ventricle (x = 4, y = 8, z = 12; x = -4, y = 8, z = 12; with 3 mm radius) as controls.

Next, the averaged time course was obtained from the seed and the correlation analysis was performed in a voxel-wise way. Contrast images were generated for each subject by estimating the regression coefficient between all brain voxels and each seed’s time series, respectively. The correlation coefficient map was then converted into a Fisher-Z map by Fisher’s r-to-z transform to improve the normality. Group analysis was later applied using the random effect model with SPM8. We first compared the rs-fc difference between MwoA patients and healthy controls using two sample t-tests. Then, we compared the changes of rs-fc difference (post-treatment minus pre-treatment) between acupuncture (verum + sham) groups (AG) and waiting-list group in factorial design module in SPM8. To explore the association between the clinical outcomes and the PAG rs-fc, we also applied regression analyses between: 1) baseline PAG rs-fc and the corresponding migraine symptom (VAS), and 2) post- and pre-treatment rs-fc changes and corresponding VAS changes in acupuncture groups. For all group analysis, disease duration, SAS, and SDS are included as non-interest covariates. A threshold of a voxel-wise P < 0.005 uncorrected and P < 0.05 family wise error (FWE) correction at cluster level was applied for all analysis.

## Results

150 patients were screened and 100 patients were recruited for this study. 46 age and sex-matched healthy controls were recruited in this study, 4 HCs were excluded due to excessive head movements. 88 patients participated in first fMRI scan, 81 patients participated in the second fMRI scan. 7 patients did not participate in the second fMRI scan, due to scheduling conflicts (2 in VA1, 2 in VA2, 1 in VA3, and 2 in SA groups). Of the 81 patients who participated in the two fMRI scans, 9 patients were excluded from data analysis due to incomplete scans (lack of resting state fMRI or T1 anatomy, 3 in V1, 1 in V2, 2 in V3, 2 in SA and 1 patient in waiting-list group) and 10 patients were excluded due to excessive head movements (1 in V1, 3 in V2, 2 in V3, 2 in SA and 2 in WT) (Fig. 1).

### Baseline characteristics

We found no statistical difference among VA1, VA2, VA3, SA and waiting-list groups in age, sex, weight, height, duration of disease, headache intensity (VAS score), headache frequency, SAS and SDS (P > 0.05). We also found no statistical difference in age, gender, weight and height between all MwoA patients and healthy controls (P > 0.05) (Table 1).

### Clinical outcomes

Compared with the baseline group, VA1, VA2, and VA3 groups showed improvement in VAS score (P < 0.05)(Table 2). SA participants showed improvement trend in VAS score (P = 0.158) (Table 2). As expected, due to relatively small sample size in each group, we found no significant difference among VA1, VA2, VA3, and SA groups in VAS score, headache frequency, SAS, and SDS improvement (P > 0.05)(Table 3), which is consistent with previous published meta-analysis reports^28^,^29^ indicating the specific effect of acupuncture treatment compared with sham treatment is only moderate. Since both verum and sham treatment reduced the VAS score in migraine patients, we merged all acupuncture groups (AG) together. We observed that AG showed significant therapeutic effects than waiting-list group in VAS score and headache frequency improvement (P < 0.05)(Table 3). Based on these clinical findings, we were able to merge all acupuncture treatment groups in rs-fc analyses to investigate the how an effective treatment can modulate PAG rs-fc.

### vlPAG rs-fc results

**Figure 3:**
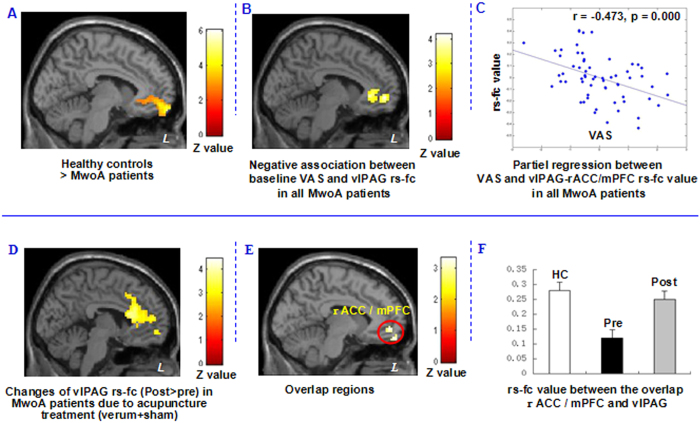
Altered resting state-functional connectivity in MwoA patients and the modulation effect of acupuncture treament (verum + sham). (**A**) Brain regions showed reduced resting state-functional connectivity with the vlPAG in MwoA patients, compared to healthy controls. (**B**) Brain regions showed reduced resting state-functional connectivity with vlPAG is associated with increased headache intensity as indicated by VAS scores in MwoA patients. (**C**) Partial regression between headache intensity and rs-fc value in MwoA patients (controlled for disease duration, SAS and SDS). (**D**) Acupuncture treatment normalized impaired vlPAG resting state-functional connectivity in MwoA patients. (**E**) Brain regions showed overlap between A and C. (**F**) The Fisher-z value of the overlap rACC in healthy controls and MwoA patients before and after acupuncture treatment respectively (mean ± SE). L, left side; MwoA, migraine without aura; rACC, rostral anterior cingulate cortex; mPFC, medial prefrontal cortex; R, right side; VAS, visual analogue scale; vlPAG, ventrolateral periaqueductal gray.

To explore the neural pathophysiology of migraine, we first applied a comparison between all MwoA patients and healthy controls using a two-sample t-test. Results indicated that compared with healthy controls, MwoA patients showed decreased rs-fc between the v1PAG and the bilateral medial prefrontal cortex (mPFC), left orbitofrontal cortex (OFC) and rostral anterior cingulate cortex (rACC), and increased rs-fc between the vlPAG and the bilateral adjacent PAG. Regression analysis indicated that the rs-fc between the vlPAG and left rACC/mPFC were negatively associated with VAS scores at baseline in MwoA patients (Fig. 3 and Table 4&5). Figure 3C shows the partial regression result between VAS and rs-fc value (peak MNI in rACC/mPFC, x = -6, y = 54, z = -3, 3 mm radius, sphere), controlled for disease duration, SAS and SDS.

We then compared post- and pre-treatment differences in all treatment groups. Results revealed that after longitudinal acupuncture treatment, MwoA patients showed increased rs-fc between vlPAG and the bilateral middle cingulate cortex (MCC) and rACC, and left mPFC (Fig. 3D and Table 4). Interestingly, this finding partially overlapped with the rs-fc difference between healthy controls and MwoA patients at rACC/mPFC.

**Figure 4:**
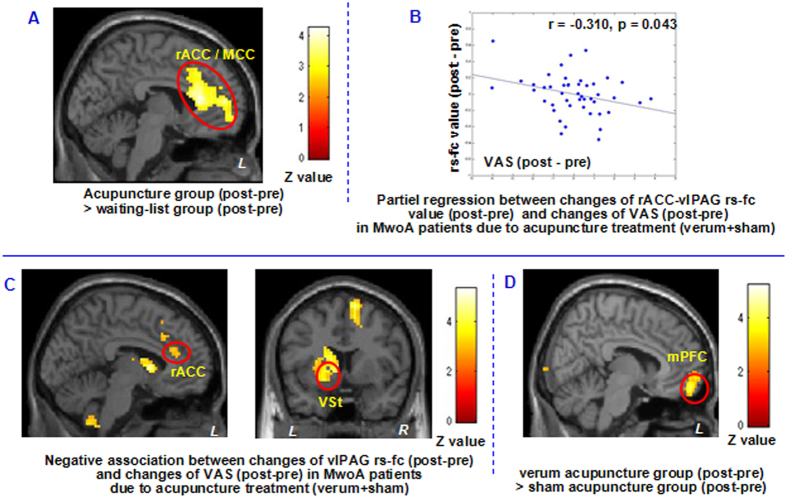
vlPAG resting state-functional connectivity comparison between acupuncture treament and waiting-list. (**A**) Brain regions showed greater PAG rs-fc increase (post- minus pre-treatment) in the acupuncture treatment group compared to waiting-list control. (**B**) Partial regression between headache intensity changes and rs-fc value changes in acupuncture group (controlled for disease duration, SAS changes and SDS changes). (**C**) Increased resting state-functional connectivity between the vlPAG and rACC/VSt after treatment was associated with reduced headache intensity in patients received acupuncture treatment. L, left side; MwoA, migraine without aura; rACC, rostral anterior cingulate cortex; R, right side; VAS, visual analogue scale; vlPAG, periaqueductal gray; VSt, ventral striatum. (**A**) Brain region showed greater PAG rs-fc increase (post- minus pre-treatment) in the verum acupuncture treatment group compared to sham acupuncture group.

To further explore the rs-fc changes at different conditions, we extracted the rs-fc Fisher-z value in the overlapping rACC/mPFC region and found that effective treatment can normalize the decreased rs-fc between the vlPAG and rACC (Fig. 3E). Regression analyses showed that changes of rs-fc (post-pre) between vlPAG and brain regions including bilateral rACC and MCC, and left superior frontal gyrus, thalamus, putamen, caudate and cerebellum, and right supplementary motor area (SMA)/preSMA and middle frontal gyrus were negatively associated with corresponding headache intensity changes (post-pre) in AG (Fig. 4 and Table 5). Figure 4B showed the partial regression result between VAS changes in acupuncture group (verum + sham acupuncture) and the corresponding rs-fc value changes (peak MNI in rACC, x = −12, y = 36, z = 21, 3 mm radius, sphere), controlled for disease duration, SAS changes and SDS changes.

In the waiting-list group, comparison between the first and second fMRI scan showed reduced rs-fc between the vlPAG and the left inferior parietal lobe, precuneus, angular gyrus. Compared with the waiting-list group, the AG treatment groups showed greater vlPAG rs-fc increases in the bilateral rACC, and left mPFC and MCC after treatments (Fig. 4A and Table 4). In addition, we also compared the difference between verum acupuncture group and sham acupuncture group. The results indicated that, compared with sham acupuncture group, verum acupuncture group showed greater vlPAG rs-fc increases with the bilateral ventral/anterior mPFC (peak MNI x = 12, y = 57, z = −18, z = 4.04, cluster size = 238), left middle occipital gyrus/cunues (peak MNI x = −21, y = −102, z = 0, z = 3.91, cluster size = 210) and right middle occipital gyrus/cunues (peak MNI x = 33, y = −99, z = 0, z = 4.15, cluster size = 348) after treatments (Fig. 4D).

As an extra control, we also ran the above analysis using a seed from ventricular aqueduct nearby the PAG seed and seeds from the forth ventricle. Using the seed ventricular aqueduct nearby the PAG, we only found that from when compared with HCs, vlPAG in MwoA patients showed increased rs-fc with the adjacent PAG (peak MNI x = 0, y = −33, z = −12, z = 4.55, cluster size = 690) at the threshold we set in rs-fc analysis; no result was found using the seeds from the forth ventricle, which further validates the findings of our experiment.

## Discussion

In this study, we investigated the PAG rs-fc changes between migraine patients and healthy controls as well as the modulation effect of the effective treatment. We found that migraine patients are associated with reduced PAG-DPMS regions rs-fc as compared with healthy controls, and the longitudinal acupuncture treatment can normalize the decreased PAG-rACC rs-fc in migraine patients. Most importantly, these changes in the rs-fc were associated with alleviation (i.e., decrease in VAS) of migraine headache intensity.

The PAG plays a central role in DPMS and is closely associated with opioid analgesia^27^. Studies suggest that PAG receives and projects to broader cortical and sub-cortical regions including the mPFC, vlPFC, ACC, precentral, postcentral, amygdala, insula, and RVM^30^,^31^,^32^. In a previous study, we found that vlPAG was functionally connected with the ACC, insula, RVM, and other regions in healthy subjects using the same seed^12^. In the present study, we found reduced vlPAG rs-fc with DPMS regions including, rACC, mPFC and OFC^33^ in MwoA patients as compared with healthy controls. Furthermore, the reduced rs-fc between vlPAG and rACC and adjunct mPFC was significantly associated with migraine intensity increase. This result is consistent with other studies reporting that reduced rs-fc between the PAG and DPMS regions (PFC, ACC, amygdala, and medial thalamus) was associated with increased frequency of migraine attacks^15^ and that gray matter (volume/thickness) decreases in pain suppression regions, such as the ACC, OFC, insula, and dorsal pons in many chronic pain conditions^34^. In addition, we also found increased rs-fc between the vlPAG and mPFC/rACC in chronic low back pain patients when these patients were experiencing endogenous chronic low back pain^13^. In this study, patients were free from migraine during scan, and we found reduced rs-fc between the PAG and mPFC/rACC. The different directions of PAG rs-fc change (increase vs decrease) imply that the PAG rs-fc may vary at different states (experiencing pain vs not experiencing pain) in chronic pain patients. Further study is needed on the dynamic changes of PAG rs-fc at different stages of migraine.

Using resting state BOLD-fMRI and PET-CT (^18^F-FDG), investigators found that affective, sensorimotor, and cognitive brain regions are involved in acupuncture treatment of migraine patients^35^,^36^,^37^,^38^. Recently, we also found that longitudinal acupuncture can significantly modulate 1) the rs-fc between PAG and mPFC^39^; 2) the rs-fc between the right frontoparietal network and the executive control network rACC)/mPFC^40^; and 3) rs-fc between the posterior mPFC and the rACC and ventral striatum^41^ in knee osteoarthritis patients. In this study, we found that acupuncture treatment can significantly modulate (normalize) impaired rs-fc, particularly the PAG- rACC/mPFC in MwoA patients. The rACC/mPFC is a rich opioid receptor area^42^, and a key region of the endogenous opioid system. Previous studies found that rACC/mPFC is actively involved in endogenous pain control, including deep brain stimulation^43^, anticipation of pain^44^, placebo^45^,^46^, attention^47^ and distraction^48^. Our results provide direct evidence suggesting that acupuncture (real and sham) may relieve migraine symptoms through the endogenous opioid DPMS.

Although there is no significant difference on clinical outcomes, we found that compared with sham acupuncture, verum acupuncture showed greater vlPAG rs-fc increases with the bilateral ventral mPFC, which is an important brain region of DPMS. This result is consistent with our previous findings that showed longitudinal verum acupuncture treatment could enhance mPFC resting state functional connectivity, compared with longitudinal sham acupuncture treatment in knee osteoarthrosis patients^40^,^41^. However the sample size of sham acupuncture group in this study is small, further studies with larger sample size are needed to address this point.

We also observed that the pre- and post-treatment rs-fc change between the PAG and the reward system (including nucleus accumbens/ventral striatum, putamen and left caudate/dorsal striatum)^49^ is significantly associated with migraine headache intensity relief. A previous study found that acupuncture needle associated with treatment context can produce greater fMRI signal increase at ventral striatum as compared to pure needle stimulation that is associated with treatment context^50^. In another study, investigator found that pain relief also produces negative reinforcement through activation of the mesocorticolimbic reward-valuation circuitry^51^. A recent human fMRI-PET study found endogenous opioid releases in the nucleus accumbens during pressure pain^52^. Studies also revealed that baseline brain response to pain stimuli at reward areas such as the nucleus accumbens can be predicted following opioid analgesia^53^. Taken together, our results demonstrate that the reward system may also play an important role in acupuncture treatment of migraine. However, since we combined both real and sham acupuncture in this study, further study is needed to explore the role of reward system in specific effect of verum acupuncture treatment.

There are several potential limitations in this study. 1) Sample size in each group was relatively small, which prevented us from testing the clinical outcome differences between different treatment groups. 2) Dropout rate in each group was relatively high, however, we would like to emphasize that the reasons for dropout do not seem to be associated with treatment response. Also, the aim of this study was to explore if an effective treatment could modulate the PAG rs-fc in migraine patients rather than to test the efficacy of acupuncture treatment. 3) We did not quantitatively record intensity of ‘deqi’ sensation, which is thought to be an important contributor to acupuncture effect^21^. 4) Our fMRI data was not acquired with cardiac-gating^54^ which minimizes physiological motion artifact due to pressure wave pulsatility in arteries within and around the brain and we did not record respiratory and heart rate data to correct for potential physiological and motion artifact. Nevertheless, the motion and CSF and white matter signal were all regressed out in data preprocess. Most importantly, we would like to argue that the potential physiological/motion influence should not be region specific as demonstrated in this study, and the lack of significant results of our control seed in the ventricle further validates our finding. This method has also been used by researchers from both our group and other groups who have achieved significant findings^13^,^14^,^15^,^16^, we thus believe that our result is meaningful. Future studies may be needed to compare the fMRI data with and without cardiac gating and physiological correction. 5) In this study, 8-Channel phased array coil was used for fMRI data acquisition. In a previous study, investigators compare signal detectability within and between multichannel brain coils (a 32-Channel and a 12-Channel) at 3.0T, they found that 32-Channel array coil can provide both detailed functional connectivity maps and reduce acquisition time^55^, which could be applied in further study. 6) Scrubbing might affect the temporal structure of the data set, nevertheless, we observed similar result when we re-analyzed the data without applying scrubbing, other methods such as the artifactual time points may be considered in future study.

## Conclusion

We found that impairment of the DPMS is associated with neural pathophysiology of migraine during interictal period. Effective treatment can normalize the reduced rs-fc between the PAG and rACC/mPFC in migraine patients, which is associated with reduction of migraine intensity. Our result demonstrated the potential role of DPMS in monitoring the development of migraine.

## Additional Information

**How to cite this article**: Li, Z. *et al*. Altered periaqueductal gray resting state functional connectivity in migraine and the modulation effect of treatment. *Sci. Rep*. **6**, 20298; doi: 10.1038/srep20298 (2016).

## Figures and Tables

**Table 1:** Baseline characteristics of MwoA patients (subjects completed two MRI scans with completed MRI data) in different group and healthy controls.

Characteristics	VA1, n = 11	VA2, n = 11	VA3, n = 13	SA, n = 11	WT, n = 16	P value *	MwoA, n = 62	HC, n = 42	P value **
Female n(%)	9 (81.8%)	8 (72.7%)	10 (77.2%)	9 (81.8%)	12 (77.5%)	0.979	48 (77.4%)	34 (81.0%)	0.808
Age (y) Mean (95%CI)	21.73 (20.56; 22.89)	21.18 (19.69; 22.67)	21.00 (19.98; 22.02)	21.18 (20.52; 21.84)	21.38 (20.33; 22.42)	0.889	21.29 (20.85; 21.73)	21.21 (20.93; 21.49)	0.771
Height (cm) Mean (95%CI)	158.00 (153.65; 162.35)	163.45 (158.25; 168.67)	159.69 (155.35; 164.04)	157.00 (154.00; 160.00)	162.63 (157.60; 167.65)	0.163	160.34 (158.40; 162.28)	161.00 (159.23; 162.77)	0.633
Weight (kg) Mean (95%CI)	51.64 (47.22; 56.05)	56.27 (48.61; 63.93)	50.73 (47.94; 53.52)	48.27 (45.91; 50.64)	53.94 (48.58; 59.29)	0.164	52.27 (50.19; 54.34)	50.98 (49.12; 52.84)	0.382
Duration (mo) Mean (95%CI)	62.91 (43.91; 81.84)	68.91 (39.88; 97.93)	67.38 (45.44; 89.33)	58.00 (39.94; 76.06)	73.31 (53.15; 93.47)	0.843	–	–	–
Headache intensity Mean (95%CI)	5.45 (4.54; 6.37)	5.32 (4.68; 5.96)	5.69 (5.12; 6.26)	5.18 (4.19; 6.17)	5.66 (5.16; 6.15)	0.765	–	–	–
Headache frequency Mean (95%CI)	5.91 (3.88; 7.93)	7.45 (5.03; 9.88)	5.54 (3.35; 7.72)	6.45 (4.34; 8.57)	4.31 (2.91; 5.71)	0.152	–	–	–
SAS score Mean (95%CI)	45.05 (39.72; 50.37)	45.27 (39.71; 50.83)	46.75 (40.50; 53.00)	45.68 (40.35; 51.01)	46.81 (41.52; 52.11)	0.980	–	–	–
SDS score Mean (95%CI)	42.41 (34.55; 50.27)	50.86 (45.14; 56.59)	43.90 (36.43; 51.38)	44.27 (37.41; 51.14)	47.34 (42.48; 52.21)	0.326	–	–	–

HC, healthy controls; MwoA, migraine without aura; VA, verum acupuncture; SA, sham acupuncture group; SAS, self-rating anxiety scale; SDS, self-rating depression scale; WT, waiting-list; *, χ^2^ test was applied for gender comparison, one-way ANOVA was applied for the rest comparisions, among VA1, VA2, VA3, SA and WT groups; **, χ^2^ test was applied for gender comparison, two-sample t test was applied for the rest comparisions, between MwoA and HC.

**Table 2:** Therapeutic effect in different groups.

Outcome measures	VA1, n = 11	VA2, n = 11	VA3, n = 13	SA, n = 11	WT, n = 16	AG, n = 46
Headache intensity Mean (95%CI)						
Baseline	5.45 (4.54; 6.37)	5.32 (4.68; 5.96)	5.69 (5.12; 6.26)	5.18 (4.19; 6.17)	5.66 (5.16; 6.15)	5.42 (5.08; 5.77)
End of treatment	3.18 (2.24; 4.12)	3.73 (2.82; 4.63)	3.15 (2.31; 3.99)	4.04 (3.09; 5.00)	5.72 (4.84; 6.59)	3.51 (3.10;3.93)
P value	0.003	0.002	0.000	0.158	0.861	0.000
Headache frequency Mean (95%CI)						
Baseline	5.91 (3.88; 7.93)	7.45 (5.03; 9.88)	5.54 (3.35; 7.72)	6.45 (4.34; 8.57)	4.31 (2.91; 5.71)	6.30 (5.31; 7.30)
End of treatment	4.18 (2.60; 5.77)	6.18 (4.13; 8.03)	4.08 (2.40; 5.76)	6.45 (3.97; 8.94)	8.63 (6.14; 11.11)	5.17 (4.27; 6.08)
P value	0.029	0.111	0.038	0.999	0.000	0.009
SAS score Mean (95%CI)						
Baseline	45.05 (39.72; 50.37)	45.27 (39.71; 50.83)	46.75 (40.50; 53.00)	45.68 (40.35; 51.01)	46.81 (41.52; 52.11)	45.73 (43.21; 48.26)
End of treatment	39.20 (31.93; 46.48)	41.77 (36.43; 47.11)	37.96 (31.68; 44.25)	38.02 (32.97; 43.08)	41.64 (37.76; 45.52)	39.18 (36.46; 41.91)
P value	0.101	0.180	0.015	0.081	0.040	0.000
SDS score Mean (95%CI)						
Baseline	42.41 (34.55; 50.27)	50.86 (45.14; 56.59)	43.90 (36.43; 51.38)	44.27 (37.41; 51.14)	47.34 (42.48; 52.21)	45.30 (42.03; 48.57)
End of treatment	41.93 (33.11; 50.75)	43.86 (36.67; 51.05)	38.92 (32.31; 45.53)	37.34 (30.69; 43.98)	41.25 (35.99; 46.51)	40.45 (37.14; 43.75)
P value	0.832	0.071	0.070	0.118	0.065	0.003

AG, acupuncture group, verum acupuncture + sham acupuncture; VA, verum acupuncture; SA, sham acupuncture group; SAS, self-rating anxiety scale; SDS, self-rating depression scale; WT, waiting list. Pair-t test was applied for comparisons in each group.

**Table 3:** Comparisons of the therapeutic effects between different groups.

Outcome measures	VA1, n = 11	VA2, n = 11	VA3, n = 13	SA, n = 11	P value *	WT, n = 16	AG, n = 46	P value **
Headache intensity Mean (95%CI)								
End of treatment	3.18 (2.24; 4.12)	3.73 (2.82; 4.63)	3.15 (2.31; 3.99)	4.04 (3.09; 5.00)	0.351	5.72 (4.84; 6.59)	3.51 (3.10;3.93)	0.000
End-baseline	−2.27 (−3.55; −0.99)	−1.59 (−2.42; −0.75)	−2.54 (−3.41; −1.66)	−1.14 (−2.80; 0.52)	0.242	0.06 (−0.68; 0.81)	−1.91 (−2.46; −1.37)	0.000
Headache frequency Mean (95%CI)								
End of treatment	4.18 (2.60; 5.77)	6.18 (4.13; 8.03)	4.08 (2.40; 5.76)	6.45 (3.97; 8.94)	0.107	8.63 (6.14; 11.11)	5.17 (4.27; 6.08)	0.012
End-baseline	−1.73 (−3.23; −0.22)	−1.27 (−2.89; 0.35)	−1.46 (−2.83; −0.10)	0.00 (−2.75; 2.75)	0.493	4.31 (2.26; 6.37)	−1.13 (−1.97; −0.29)	0.000
SAS score Mean (95%CI)								
End of treatment	39.20 (31.93; 46.48)	41.77 (36.43; 47.11)	37.96 (31.68; 44.25)	38.02 (32.97; 43.08)	0.745	41.64 (37.76; 45.52)	39.18 (36.46; 41.91)	0.336
End-baseline	−5.84 (−13.05; 1.37)	−3.50 (−8.91; 1.91)	−8.79 (−15.54; −2.04)	−7.66 (−16.46; 1.14)	0.670	−5.17 (−10.07; −0.27)	−6.55 (−9.74; −3.36)	0.649
SDS score Mean (95%CI)								
End of treatment	41.93 (33.11; 50.75)	43.86 (36.67; 51.05)	38.92 (32.31; 45.53)	37.34 (30.69; 43.98)	0.520	41.25 (35.99; 46.51)	40.45 (37.14; 43.75)	0.799
End-baseline	−0.48 (−5.35; 4.40)	−7.00 (−14.72; 0.72)	−4.98 (−10.44; 0.48)	−6.93 (−15.95; 2.09)	0.432	−6.09 (−12.61; 0.42)	−4.85 (−7.96; −1.74)	0.697

AG, acupuncture groups; HC, healthy controls; VA, verum acupuncture; SA, sham acupuncture; SAS, self-rating anxiety scale; SDS, self-rating depression scale; WT, waiting list; *, one-way ANOVA was applied for the comparisions among VA1, VA2, VA3, SA and WT groups; **, two-sample t test was applied for the comparisions between MwoA and HC.

**Table 4:** The vlPAG resting state functional connectivity in MwoA patients and the changes in different groups.

vlPAG resting state-functional connectivity difference between MwoA patients in baseline and healthy controls							Changes of vlPAG resting state-functional connectivity in MwoA patients due to acupuncture treatment (verum + sham)						
Contrast	Voxels	Brain Region	MNI (x, y, z)			Z	Contrast	Voxels	Brain Region	MNI (x, y, z)			Z
HC>MwoA	297	L OFC	−3	63	−18	5.81	Post>Pre	408	L MCC	−3	27	24	3.51
		L mPFC	−6	54	−12	3.91			L mPFC	−9	51	−6	3.18
		L rACC	−12	48	3	2.84			L rACC	−12	51	9	3.63
		R mPFC	6	57	−9	3.41			R MCC	3	18	15	4.29
	196	L OFC	−36	21	−21	4.34			R rACC	3	39	15	3.31
MwoA>HC	236	Bilateral PAG	3	−30	−15	3.55	Pre >Post		No brain region above the threshold				
**Difference between acupuncture treatment group (AG) and WT group in the changes of vlPAG resting state-functional connectivity**							**Changes of vlPAG resting state-functional connectivity in MwoA patients in WT group**						
AG >WT	501	L MCC	0	27	12	4.11	Post>Pre		No brain region above the threshold				
		L mPFC	−6	54	−6	3.24	Pre>Post	221	L Inf Parietal Lob	−39	−45	42	3.74
		L rACC	−12	51	6	3.98			L Precuneus	−33	−63	45	3.72
		R rACC	6	39	15	3.58			L Angular gyrus	−33	−57	39	3.29
WT>AG		No brain region above the threshold											

AG, acupuncture treatment group; L, left side; MCC, middle cingulate cortex; mPFC, medial prefrontal cortex; MwoA, migraine without aura; OFC, orbitofrontal cortex; rACC, rostral anterior cingulate cortex; R, right side; SMA, supplementary motor area; VA, verum acupuncture; VAS, visual analogue scale; vlPAG, ventrolateral periaqueductal gray; WT, waiting-list.

**Table 5:** The association between vlPAG resting state-functional connectivity and VAS.

Association between baseline VAS and baseline vlPAG resting state-functional connectivity in all MwoA patients							Association between VAS changes and vlPAG resting state-functional connectivity changes due to acupuncture treatment (verum + sham)						
Contrast	Voxels	Brain Region	MNI (x, y, z)			Z	Contrast	Voxels	Brain Region	MNI (x, y, z)			Z
Negative	226	L mPFC/rACC	−6	54	−3	3.73	Negative	523	L MCC/rACC	−12	36	21	3.13
									L Sup Frontal gyrus	−24	42	27	3.77
									R MCC/rACC	15	48	21	3.88
									R SMA/preSMA	12	9	66	4.21
									R Mid Frontal gyrus	27	42	27	3.76
								296	L Putamen	−18	15	0	4.71
									L Caudate	−9	0	15	4.57
									L Thalamus	−15	−12	9	3.57
								300	L Cerebellum	−9	−57	−54	4.25
Positive	No brain region above the threshold						Positive	No brain region above the threshold					

L, left side; MCC, middle cingulate cortex; mPFC, medial prefrontal cortex; MwoA, migraine without aura; rACC, rostral anterior cingulate cortex; R, right side; SMA, supplementary motor area; VAS, visual analogue scale; vlPAG, ventrolateral periaqueductal gray.
